# Supplementary material for: Understanding the Holobiont: Crosstalk Between Gut Microbiota and Mitochondria During Long Exercise in Horse
Source: Front Mol Biosci. 2021 Apr 8;8:656204. doi: 10.3389/fmolb.2021.656204 (PMC8063112; doi:10.3389/fmolb.2021.656204)
Supplement: Supplementary file 16 [file table15.docx]

Supplementary Material

# Supplementary Information

**Creation of a consensus list for literature-based meta-analytical analysis of mitochondrial-related genes.**

We retrieved four gene lists: (1) the Integrated Mitochondrial Protein Index (IMPI) gene list (Smith and Robinson, 2019). Only the human gene list (‘Human IMPI genes’, ‘MitoMiner version Q2 2018’, downloaded from http://mitominer.mrc-mbu.cam.ac.uk/release-4.0/impi.do) was retained (1,626 genes) to facilitate finding horse/human orthologs; (2) the Mitocarta Inventory (Calvo et al., 2016) gene list. As before, only the human gene list (‘Human MitoCarta 2.0 genes’, ‘MitoCarta 2.0 genes in MitoMiner’ downloaded from http://mitominer.mrc-mbu.cam.ac.uk/release-4.0/mitocarta.do) was retained (1,158 genes) to facilitate the identification of horse/human orthologs; (3) all of the 618 genes included in the following KEGG pathways: hsa00020, hsa00061, hsa00062, hsa00071, hsa00072, hsa00100, hsa00130, hsa00140, hsa00190, hsa00240, hsa00280, hsa00290, hsa00471, hsa00480, hsa00628, hsa00760, hsa00860, hsa01212, hsa04020, hsa04024, hsa04072, hsa04115, hsa04136, hsa04137, hsa04140, hsa04210, hsa04215, hsa04350, hsa04370, hsa04668 and hsa04979. The genes in each KEGG pathway were retrieved from the KEGG database (release 96.0) using the R package KEGGREST; (4) a custom list including 103 genes found in the literature (Bianchessi et al., 2016; Cosson et al., 2012; Gustafsson et al., 2016; Gustafsson and Samuelsson, 2001; Lee et al., 2015; Nicholls and Gustafsson, 2018; Pearce et al., 2017; Rizzuto et al., 2012; Wang et al., 2016). These four gene sets were merged to create a consensus list, which included 2,082 unique genes (Supplementary Table S2).

# Supplementary Figures and Tables

## Supplementary Figures


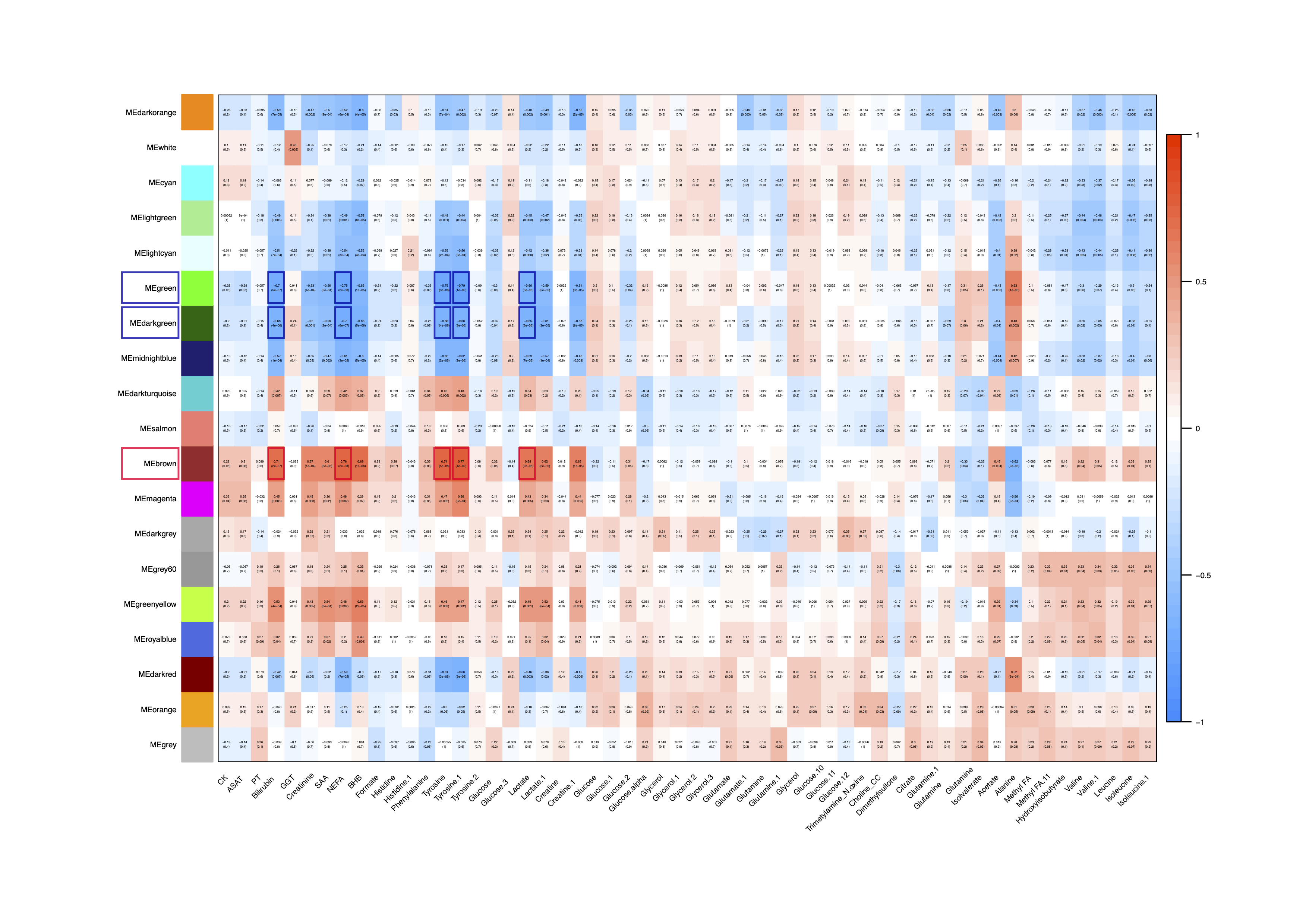


**Supplementary Figure 1.** **Correlations between eigengene modules and metabolites from WGCNA method.** Each module is labelled with a unique colour as an identifier. A module is considered positively (highlighted in red) or negatively (highlighted in blue) associated to metabolites if the Pearson *r* correlation values were ≥ |0.65| for at least 5 molecules (highlighted again in red or blue) and if the corresponding *p*-values are ≤ 1e-05. Within each cell, upper values indicate the correlation values between modules and metabolites, while lower values are the corresponding *p*-values.

**
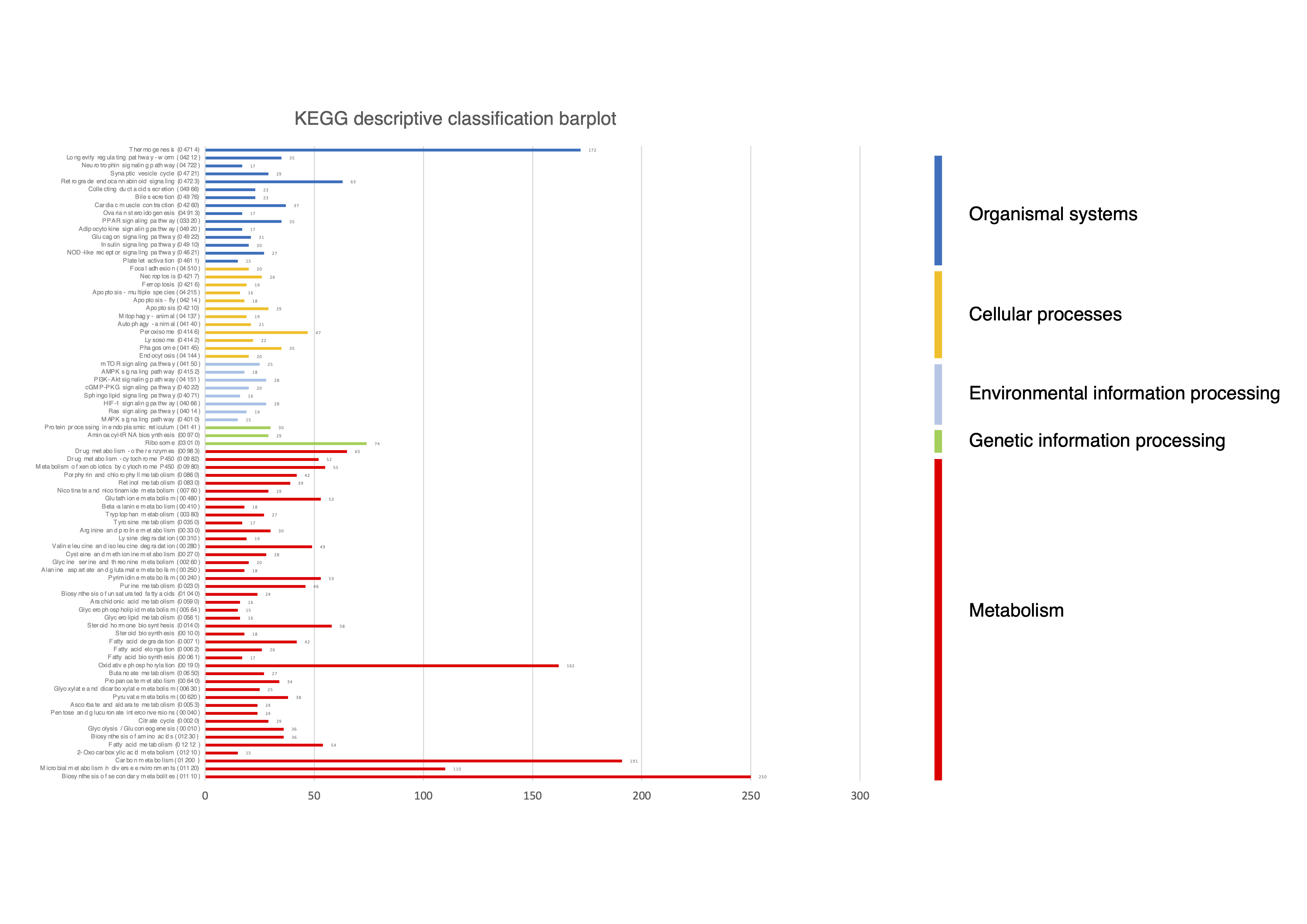
**

**Supplementary Figure 2. Descriptive KEGG pathway classification bar plot obtained using the consensus list of genes related to mitochondria.**

The horizontal bars represent the absolute number of genes found in third-level KEGG pathways, grouped in first-level KEGG pathways using a colour code. The vertical bars on the right indicate the names of first-level pathways.

**
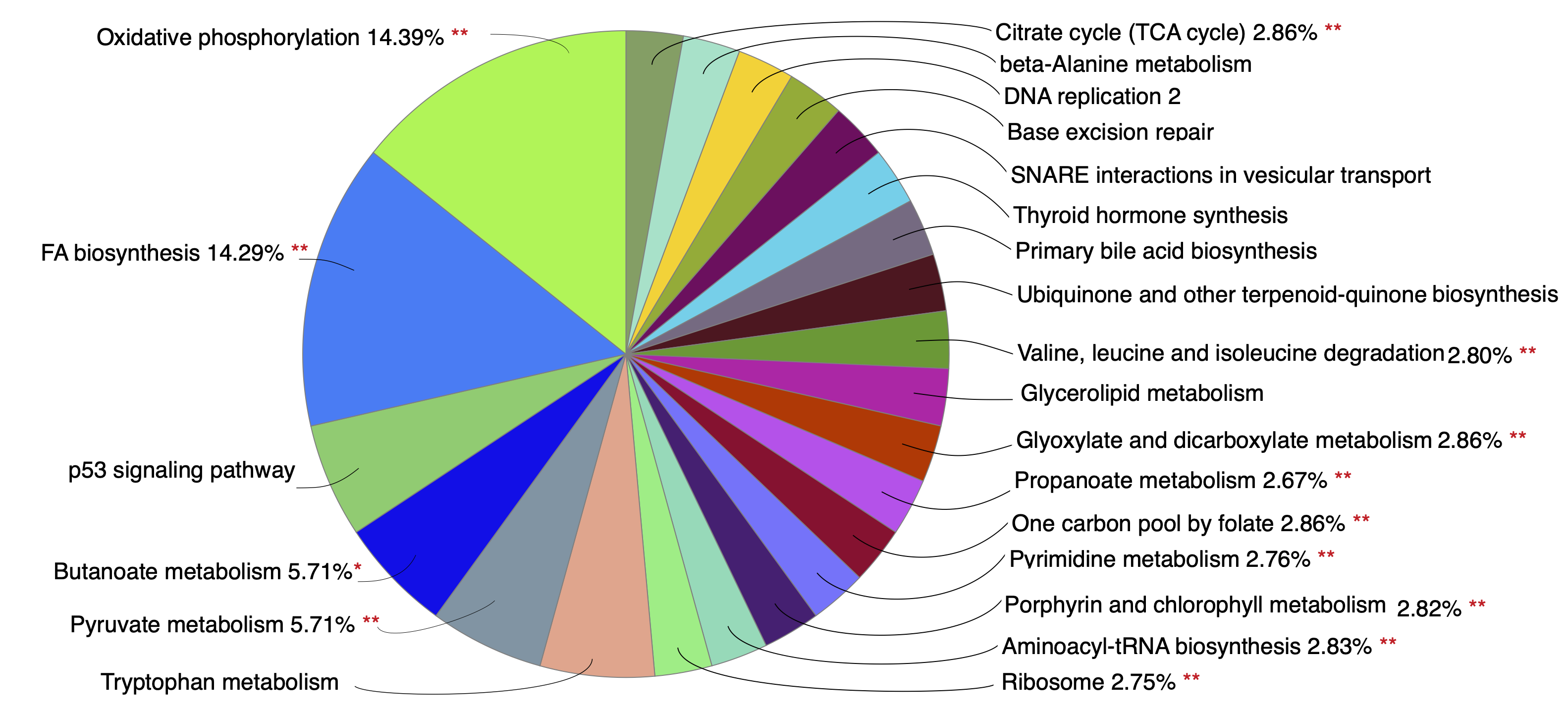
**

**Supplementary Figure 3. Functional classification of the mt-related genes.**

Functional classification of mt-related genes obtained with ClueGO. The chart shows the functional groups found as enriched, and the name of the group is represented by the group leading term. The significant enriched functional groups are marked with a red *.

**
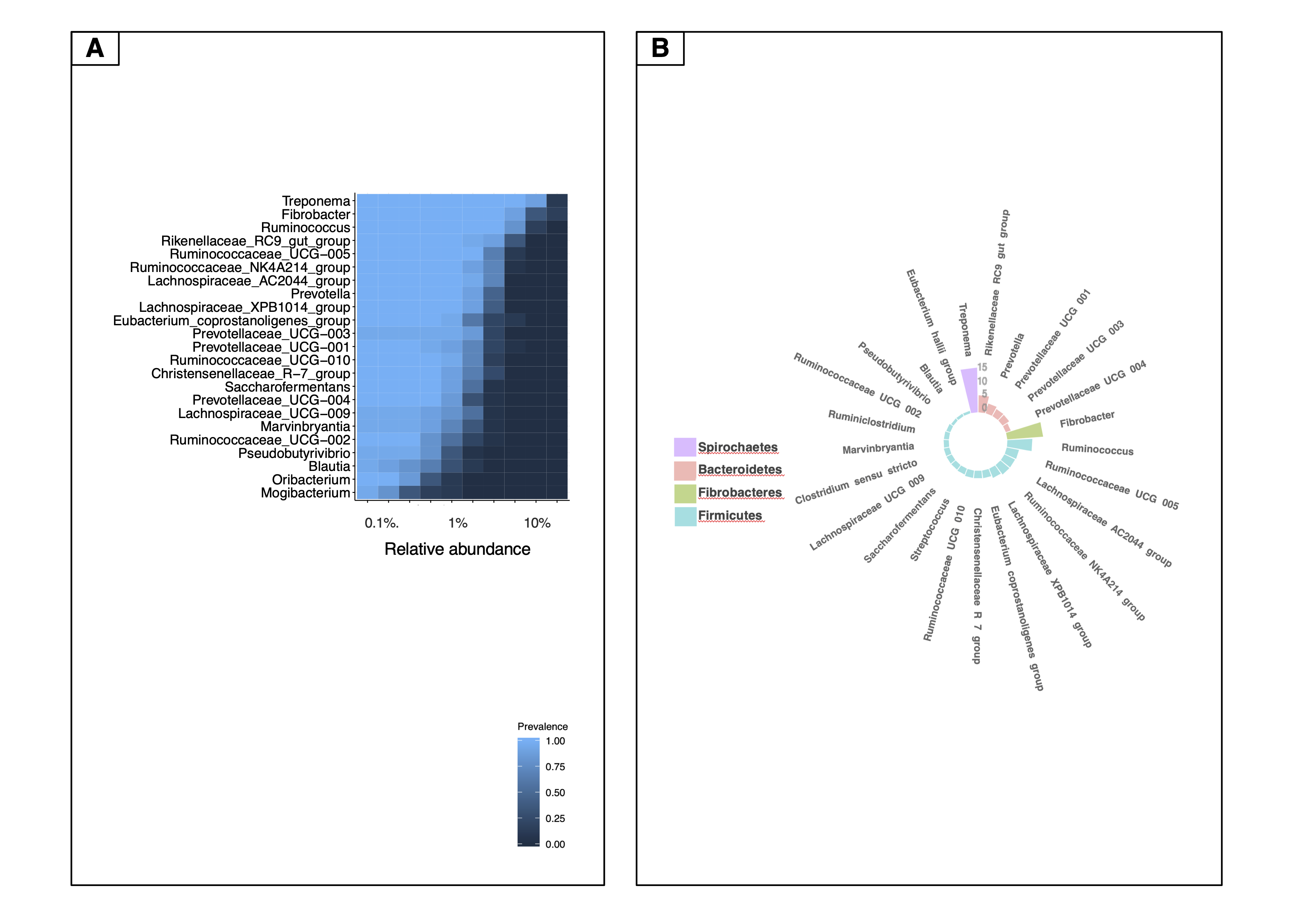
**

**Supplementary Figure 4. Composition of the core microbiota.**

A heatmap showing the core microbiota and its prevalence at different detection thresholds. Only the genera shared by 99% of individuals in the cohort and with a minimum detection threshold of 0.1% are shown.

B Circular stacked barplot of the main genera included in the core genome. Genera are coloured according to the phylum they belong to.

## Supplementary Tables

**Supplementary Table S1 - Metadata of the horses recruited in the experiment.**

The letters between brackets in the “Ranking” column indicate the causes of the elimination of the horse: “L” corresponds to “lameness”, R” to “retired” and “M” to “metabolic issues”.

**Supplementary Table S2 -** **Consensus list of genes related to mitochondria obtained as described in the Supplementary Information.**

Gene description, gene localizations and gene types were determined according to IPA (https://www.qiagenbioinformatics.com/products/ingenuity-pathway-analysis).

**Supplementary Table S3 - Relative abundance of metabolites obtained from blood of the 20 horses under study collected before and after the endurance ride.**

**Supplementary Table S4 - Biochemical parameters obtained from the blood of the 20 horses under study collected before and after the endurance race.**

**Supplementary Table S5 - Fecal pH and fecal short chain fatty acids measurements in the 20 horses under study before the endurance race.**

**Supplementary Table S6 - ASV taxonomical assignments and ASV counts for the 20 horses under study**.

**Supplementary Table S7 - Non-normalized annotated abundance genera table observed in the fecal samples of the 20 horses under study (“G1”).**

**Supplementary Table S8 - Concentrations of bacteria, ciliate protozoa and anaerobic fungi in the feces of the 20 horses under study.**

**Supplementary Table S9 - Genera table obtained using the mixMC framework (“G2”)**.

Genera with less than 1% counts with respect to the total number were removed, and subsequently a centered

**Supplementary Table S10 - List of genes found differentially expressed in whole blood in T1 with respect to T0 horses.**

Columns two and three show the mean log_2_ normalized expression values in the two time points, while columns four and five show the log_2_ FC values and the Bonferroni adjusted *p*-values. Columns six to nine indicate the genes that are also found in the mt-related consensus list. The gene lists used to create the final consensus are indicated separately.

**Supplementary Table S11 -** **List of genes included in each of the three eigengene modules found as correlated to metabolites using WGCNA.**

The modules are indicated using the default WGCNA naming convention, and highlighted in blue to indicate negative correlation and red to indicate positive correlation.

**Supplementary Table S12 - List of mt-related genes, defined as the intersection between the -** **consensus list of genes related to mitochondria and the set of differentially expressed genes.**

Gene description, gene localizations and gene types were determined according to IPA (<https://www.qiagenbioinformatics.com/products/ingenuity-pathway-analysis>). Columns from N from AG show the expression values obtained by subtracting the T0 from the T1 expression matrix values, i.e., by calculating the ratio between T1 and T0 log scaled expression values from the two matrices.

**Supplementary Table S13 - Correlated variables obtained using DIABLO on all of the available data sets.**

Gene descriptions and localizations were determined using the IPA database (https://www.qiagenbioinformatics.com/products/ingenuity-pathway-analysis). Molecular pathways were determined using ClueGO 2.5.7 in the case of genes.

**Supplementary Table S14 -** **Correlation matrix of the associations between mt-related genes and bacterial genera obtained using the rCCA method.**

Only the genes and the genera for which at least one association value data point presented *r* ≥ |0.55| are shown.

**3. Supplementary Data sheets**

**Supplementary Data sheet 1.** **ASV** **FASTA** **file.**

Fasta file containing the 3,385 unique ASV. The header contains the name of the ASV (used for a unique description of the sequence) and its assigned taxonomy (family and genus level). The first line is followed by the sequence itself in standard one-letter character string.

## Supplementary References

Bianchessi, V., Vinci, M. C., Nigro, P., Rizzi, V., Farina, F., Capogrossi, M. C., et al. (2016). Methylation profiling by bisulfite sequencing analysis of the mtDNA Non-Coding Region in replicative and senescent Endothelial Cells. *Mitochondrion* 27, 40–47. doi:10.1016/j.mito.2016.02.004.

Calvo, S. E., Clauser, K. R., and Mootha, V. K. (2016). MitoCarta2.0: An updated inventory of mammalian mitochondrial proteins. *Nucleic Acids Res.* 44, D1251–D1257. doi:10.1093/nar/gkv1003.

Cosson, P., Marchetti, A., Ravazzola, M., and Orci, L. (2012). Mitofusin-2 Independent Juxtaposition of Endoplasmic Reticulum and Mitochondria: An Ultrastructural Study. *PLoS One* 7, e46293. doi:10.1371/journal.pone.0046293.

Gustafsson, C. M., Falkenberg, M., and Larsson, N. G. (2016). Maintenance and Expression of Mammalian Mitochondrial DNA. *Annu. Rev. Biochem.* 85, 133–160. doi:10.1146/annurev-biochem-060815-014402.

Gustafsson, C. M., and Samuelsson, T. (2001). Mediator - A universal complex in transcriptional regulation. *Mol. Microbiol.* 41, 1–8. doi:10.1046/j.1365-2958.2001.02481.x.

Lee, C., Zeng, J., Drew, B. G., Sallam, T., Martin-Montalvo, A., Wan, J., et al. (2015). The mitochondrial-derived peptide MOTS-c promotes metabolic homeostasis and reduces obesity and insulin resistance. *Cell Metab.* 21, 443–454. doi:10.1016/j.cmet.2015.02.009.

Nicholls, T. J., and Gustafsson, C. M. (2018). Separating and Segregating the Human Mitochondrial Genome. *Trends Biochem. Sci.* 43, 869–881. doi:10.1016/j.tibs.2018.08.007.

Pearce, S. F., Rebelo-Guiomar, P., D’Souza, A. R., Powell, C. A., Van Haute, L., and Minczuk, M. (2017). Regulation of Mammalian Mitochondrial Gene Expression: Recent Advances. *Trends Biochem. Sci.* 42, 625–639. doi:10.1016/j.tibs.2017.02.003.

Rizzuto, R., De Stefani, D., Raffaello, A., and Mammucari, C. (2012). Mitochondria as sensors and regulators of calcium signalling. *Nat. Rev. Mol. Cell Biol.* 13, 566–578. doi:10.1038/nrm3412.

Smith, A. C., and Robinson, A. J. (2019). Mitominer v4.0: An updated database of mitochondrial localization evidence, phenotypes and diseases. *Nucleic Acids Res.* 47, D1225–D1228. doi:10.1093/nar/gky1072.

Wang, S., Jacquemyn, J., Murru, S., Martinelli, P., Barth, E., Langer, T., et al. (2016). The Mitochondrial m-AAA Protease Prevents Demyelination and Hair Greying. *PLoS Genet.* 12, e1006463. doi:10.1371/journal.pgen.1006463.
